# Emergence of Autochthonous *Leishmania* (*Mundinia*) *martiniquensis* Infections in Horses, Czech Republic and Austria, 2019–2023

**DOI:** 10.3201/eid3109.250254

**Published:** 2025-09

**Authors:** David Modrý, Edmund K. Hainisch, Hans-Peter Fuehrer, Edwin Kniha, Maria Sophia Unterköfler, Jovana Sádlová, Petr Jahn, Kristína Řeháková, Kamil Sedlák, Jan Votýpka

**Affiliations:** Masaryk University, Brno, Czech Republic (D. Modrý); Czech University of Life Sciences, Prague, Czech Republic (D. Modrý); Biology Centre of Czech Academy of Sciences, České Budějovice, Czech Republic (D. Modrý, J. Votýpka); University of Veterinary Medicine, Vienna, Austria (E. Hainisch, H.-P. Fuehrer, M.S. Unterköfler); Medical University of Vienna, Vienna (E.K. Kniha); Charles University, Prague (J. Sádlová, J. Votýpka); University of Veterinary Sciences Brno, Brno (P. Jahn, K. Řeháková); State Veterinary Institute Prague, Prague (K. Sedlák)

**Keywords:** Leishmania, horses, vector-borne infections, parasites, zoonoses, Central Europe, Mundinia, Czech Republic, Austria

## Abstract

We report 4 cases of equine cutaneous leishmaniasis caused by *Leishmania martiniquensis* in Czech Republic and Austria, outside the known endemic range of leishmaniases. The parasite should be considered as a potential cause of cutaneous lesions in horses; the risk for zoonotic transmission to immunocompromised humans is anticipated throughout central Europe.

Leishmaniasis is a relatively rare equine disease caused by several *Leishmania* spp. protozoan parasites. In Mediterranean Europe, clinical leishmaniasis in animals (mainly domestic carnivores) and humans is primarily caused by *L. infantum*. In areas endemic for *L. infantum*, sporadic cases of leishmaniosis in horses have also been reported, typically manifesting as ulcerating cutaneous nodules (*1*). During 2002–2010, cases of leishmaniosis were reported in horses (*2*) and cattle (*3*) in areas north of the Alps, which are considered nonendemic because of the low abundance of *L. infantum* vectors. Those sporadic cases were initially attributed to *L. siamensis* but were later reclassified as *L. martiniquensis* (*4*).

*L. martiniquensis*, a member of the subgenus *Mundinia*, is a zoonotic species originally described from a human visceral case in the Caribbean (*5*). *L. martiniquensis* parasites have wide distribution, spanning >3 continents, overlapping with other *Leishmania* species in many areas, including Europe (*6*). However, the full host range and epidemiology remain unclear. The distribution of cases outside the range of *Phlebotomus*/*Lutzomyia* sand flies supported by recent experimental studies and field surveys in Thailand suggest the involvement of biting midges (*Culicoides* spp., Ceratopogonidae) in transmission (*7*–*9*).

Approximately a decade after cases of *L. martiniquenis* infection were reported in Germany and Switzerland, we present 4 independent cases of cutaneous leishmaniosis in horses outside the known range of leishmaniasis in Europe. Our report includes a phylogenetic analysis of the detected isolates and results of a pilot serologic examination.

## The Study

*L. martiniquensis* was identified in 4 sport horses during 2019–2023 (Table). Case 1 (identified in May 2019) was in a 4-year-old Akchal-Teke mare admitted to the veterinary clinic of the University of Veterinary Sciences in Brno, Czech Republic. The mare had several small nodules (3–10 mm) on the left upper eyelid; the largest was localized near the medial canthus, measuring ≈1 cm in diameter. The mare lived in north Moravia and had been imported from Ukraine 2 years previously without any obvious lesions. Equine sarcoid was suspected on the basis of clinical examination, and bovine papillomavirus type 1 was detected by PCR in the skin smear. Case 2 (identified in May 2021) was in a 5-year-old Kladruber mare from a large stud farm that was admitted to the clinic with a group of small nodules (5–15 mm) located unilaterally on the facial area near to the lower eyelid. Case 3 was in a 5-year-old Fjord mare seen in May 2021 by veterinarians at the Equine Clinic of the Veterinary University (Vienna, Austria) with nodular lesions on the lower eyelid, chest, and udder; *Leishmania* was detected in the eyelid and udder lesions and bovine papillomavirus was detected in all 3 lesions. Case 4 was in a 12-year-old gelding living in the northwestern Czech Republic, first seen by the veterinarian in January 2023 for lesions on the left facial area. The clinical manifestation was very similar to those seen in cases 1–3. Again, the lesions were initially suspected to be sarcoid tumors, but the surface eventually exulcerated into an open wound. With supportive treatment, the lesion resolved over a period of 15 months; follow-up at 27 months showed no recurrence of lesions.

**Table:** Overview of detected cases in study of autochthonous *Leishmania* (*Mundinia*) *martiniquensis* infections in horses, Czech Republic and Austria, 2019–2023*

Case no.	Geographic origin and time of first diagnostics	Lesion localization	Methods of leishmania detection
1	Olomouc district, Czech Republic, May 2019	Periorbitally above the left eye	Cytology, cultivation, ITS1 PCR
2	Pardubice district, Czech Republic, May 2021	Periorbitally under the right eye	Cytology, cultivation, ITS1 PCR
3	Styria, Graz-Umgebung district, Austria, May 2021	Lower eyelid	Cytology, histology, ITS1 and 18SrDNA PCR
4	Ústí nad Labem district, Czech Republic, Jan 2023	Periorbitally around left canthus and on conjunctiva of the left eye	Cytology, ITS1 PCR

*ITS, internal transcribed spacer.

**Figure 1:**
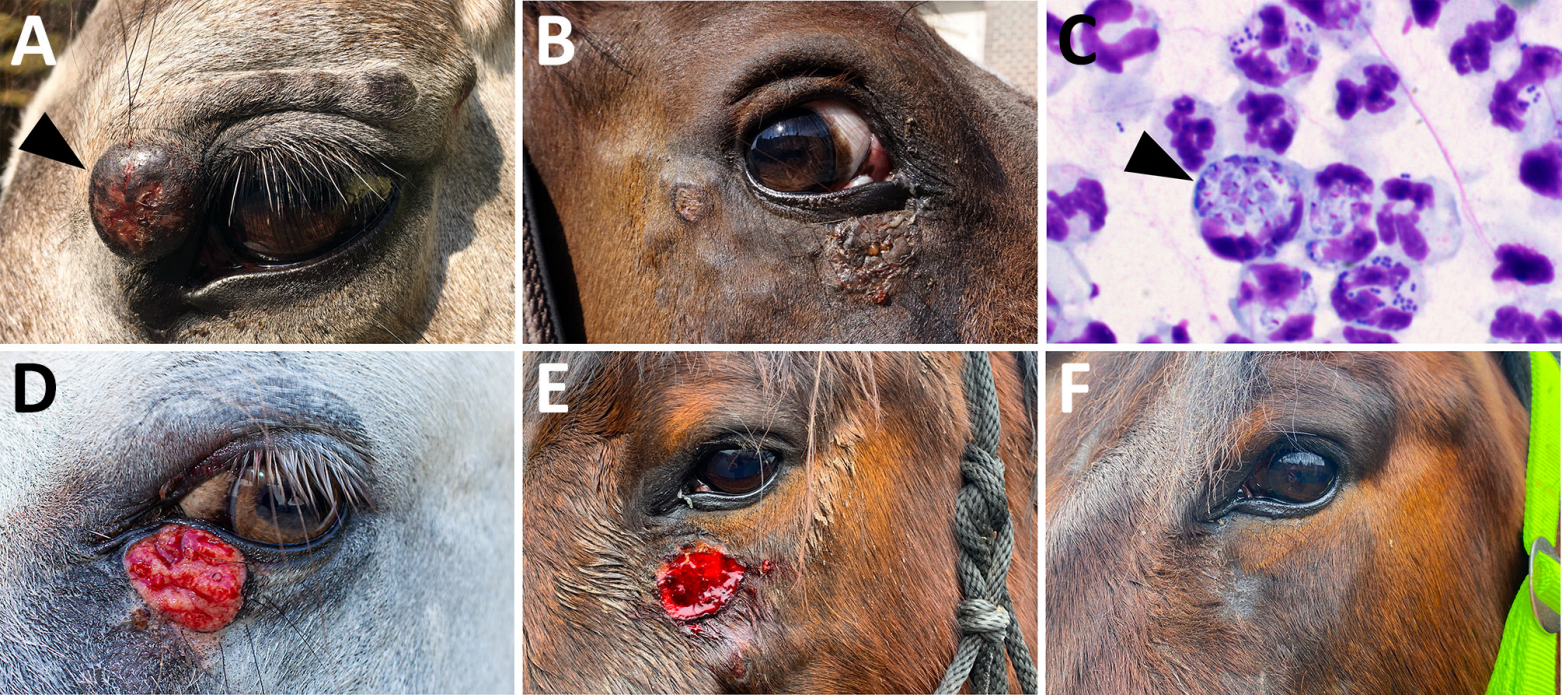
Cutaneous lesions during initial clinical examination and detection of *Leishmania* amastigotes from study of autochthonous *Leishmania* (*Mundinia*) *martiniquensis* infections in horses, Czech Republic and Austria, 2019–2023. A) Periorbital nodular lesions from case 1 (*L. martiniquensis* was cultured from a fine needle aspiration biopsy of the largest lesion, indicated by arrowhead); B) periorbital lesions in case 2; C) *Leishmania* amastigotes (indicated by arrowhead) in a stained smear from sample from case 1*;* D) lower eyelid lesion in case 3 (image by Christian Bernkopf); E) facial lesions in case 4 at the time of *Leishmania* detection; F) photograph of case 4 horse showing no recurrence 27 months later.

**Figure 2:**
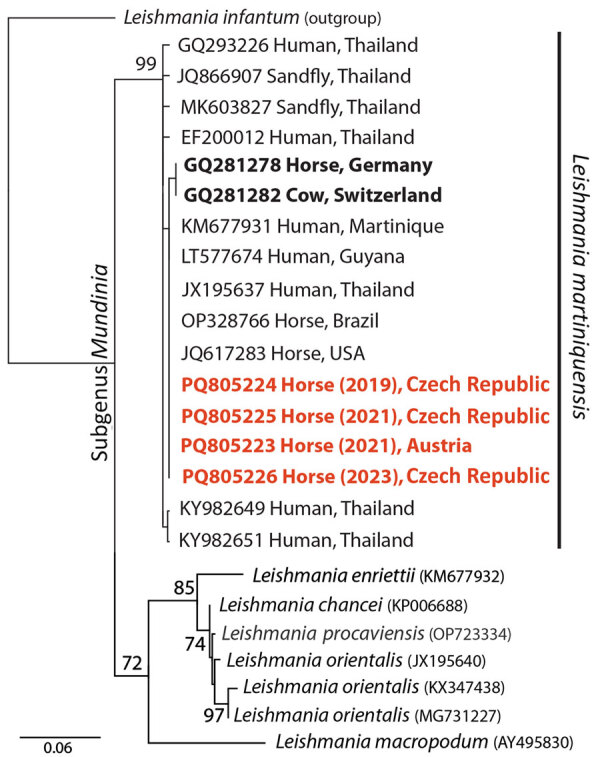
Phylogenetic analysis of isolates from study of autochthonous *Leishmania* (*Mundinia*) *martiniquensis* infections in horses, Czech Republic and Austria, 2019–2023. Analysis of the internal transcribed spacer 1 sequences was conducted using a maximum-likelihood tree with *L. infantum* as an outgroup; GenBank accession numbers precede the host and locality description. Red bold text indicates cases from this study. Black bold text indicates previous cases from Europe. Node support values were derived through bootstrapping with 1,000 replicates. Scale bar indicates number of nucleotide substitutions per site.

We obtained bioptic samples from cutaneous lesions using a fine needle aspiration biopsy (FNAB) for cases 1, 2, and 4 or as impression smears for case 3. We conducted routine microscopic evaluation of the FNAB material after Diff-Quick staining. Examination of the smears revealed intracytoplasmic *Leishmania* amastigotes in cells tentatively identified as neutrophiles (Figure 1). We cultured material obtained by FNAB from periorbital lesions (cases 1, 2, and 4) at 23°C on rabbit blood agar SNB-9 supplemented with fetal bovine serum, RPMI-1640, Schneider’s medium, and antibiotics; we then cryopreserved a single strain. Conventional PCR targeting the *Leishmania* internal transcribed spacer 1 (*10*) performed on clinical material revealed identical sequences in all 4 cases with 100% identity to other GenBank sequences of *L. martiniquensis* worldwide but only 99.5% concordance with previous cases in Germany and Switzerland (Figure 2).

Antibodies to *Leishmania* were detected by an indirect fluorescent antibody test using glass slides coated with promastigote *L. infantum* (VMRD, https://www.vmrd.com) and antihorse IgG (whole molecule) FITC conjugate (Sigma Aldrich, https://www.sigmaaldrich.com). We diluted serum samples in a 2-fold series starting with a 1:50 base dilution and used positive and negative control serum samples. We considered a titer >50 positive. We found antibodies to *Leishmania* at titers of 50 (cases 1, 2, and 3) and 100 (case 4).

## Conclusions

We report 4 equine cases of *L. martiniquensis* infection outside the known range of the typical leishmaniasis caused by *L. infantum* in Europe, detected >12 years after the last published *L. martiniquensis* cases in Germany (*2*) and Switzerland (*3*). The cases occurred over a period of >3 years with no proven link between them and were also geographically dispersed across central Europe, suggesting that horses play a nonnegligible role as reservoir hosts throughout the range of *L. martiniquensis*. The symptomatology of *L. martiniquensis* cases in horses is strikingly uniform. In all 4 newly described cases, infection was diagnosed as cutaneous lesions near the eyes or in the facial area, resembling previous instances in which 7 of 10 cases were reported as lesions on the head (*2*,*11*,*12*).

All 4 cases were initially suspected to be sarcoid, a common skin tumor in horses caused by bovine papillomaviruses types 1, 2, and 13. Of note, in 2 cases (case 1 and 3) bovine papillomavirus types 1 and 2 were detected by PCR in lesions with *Leishmania* but also in lesions without the parasite. This association between sarcoid-like lesions and *Leishmania* infection is very suggestive. We therefore believe that the sarcoid may be attractive to blood-sucking insects (including biting midges, the potential vectors of *Mundinia*), thus opening the window for parasite infection. Additional cases of *L. martiniquensis* infection could possibly be underreported because of misdiagnosis and treatment as sarcoid or masked by a true sarcoid. Also, cases of cutaneous leishmaniosis in herbivores diagnosed in Europe should always be evaluated for the possibility of being caused by *L. martiniquensis*, even in areas in which *L. infantum* is endemic (*13*), particularly in the absence of sand flies.

The serologic response to *L. martiniquensis* remains poorly understood. A single case of cutaneous leishmaniasis in a cow revealed a robust antibody response (*3*). More recently, Carbonara et al. (*13*) reported low antibody titers in equids, including those with skin lesions or asymptomatic infections. Consistent with those observations, our findings confirm that horses with mild skin lesions exhibit only a limited antibody response. Nevertheless, serologic testing during active infection could serve as a valuable diagnostic tool.

The presence of biting midges is ubiquitous in Europe (*14*), and the equine population is in daily contact with them during active season. The occurrence of 4 independent cases of equine leishmaniosis caused by *L. martiniquensis* suggests its endemic status and circulation in Central Europe. The extent of distribution of this kinetoplastid in the equine population and other hosts in Europe remains speculative, as does its transmission biology. Given its zoonotic potential, this pathogen should be widely investigated in cases of equine skin lesions using a combination of cytology and PCR followed by sequencing. Similarly, possible *L. martiniquensis* infection should be anticipated in suspected visceral and cutaneous cases of human leishmaniasis, including patients without a history of travel to endemic areas.
